# Metabolites of *Geum aleppicum* and *Sibbaldianthe bifurca*: Diversity and α-Glucosidase Inhibitory Potential

**DOI:** 10.3390/metabo13060689

**Published:** 2023-05-25

**Authors:** Nina I. Kashchenko, Daniil N. Olennikov, Nadezhda K. Chirikova

**Affiliations:** 1 Laboratory of Biomedical Research, Institute of General and Experimental Biology, Siberian Division, Russian Academy of Science, 6 Sakh’yanovoy Street, 670047 Ulan-Ude, Russia; olennikovdn@mail.ru; 2 Department of Biochemistry and Biotechnology, North-Eastern Federal University, 58 Belinsky Street, 677027 Yakutsk, Russia; hofnung@mail.ru

**Keywords:** Rosaceae, *Geum aleppicum*, *Potentilla bifurca*, avens, diabetes, flavonoids, ellagitannins, gemin A, high-performance liquid chromatography, HPLC activity-based profiling

## Abstract

α-Glucosidase inhibitors are essential in the treatment of diabetes mellitus. Plant-derived drugs are promising sources of new compounds with glucosidase-inhibiting ability. The *Geum aleppicum* Jacq. and *Sibbaldianthe bifurca* (L.) Kurtto & T.Erikss. herbs are used in many traditional medical systems to treat diabetes. In this study, metabolites of the *G. aleppicum* and *S. bifurca* herbs in active growth, flowering, and fruiting stages were investigated using high-performance liquid chromatography with photodiode array and electrospray ionization triple quadrupole mass spectrometric detection (HPLC-PDA-ESI-tQ-MS/MS). In total, 29 compounds in *G. aleppicum* and 41 components in *S. bifurca* were identified including carbohydrates, organic acids, benzoic and ellagic acid derivatives, ellagitannins, flavonoids, and triterpenoids. Gemin A, miquelianin, niga-ichigoside F1, and 3,4-dihydroxybenzoic acid 4-*O*-glucoside were the dominant compounds in the *G. aleppicum* herb, while guaiaverin, miquelianin, tellimagrandin II_2_, casuarictin, and glucose were prevailing compounds in the *S. bifurca* herb. On the basis of HPLC activity-based profiling of the *G. aleppicum* herb extract, the most pronounced inhibition of α-glucosidase was observed for gemin A and quercetin-3-*O*-glucuronide. The latter compound and quercetin-3-*O*-arabinoside demonstrated maximal inhibition of α-glucosidase in the *S. bifurca* herb extract. The obtained results confirm the prospects of using these plant compounds as possible sources of hypoglycemic nutraceuticals.

## 1. Introduction

Glycemic control is an essential therapy for patients with diabetes mellitus. Monitoring of postprandial hyperglycemia by inhibiting carbohydrate hydrolases (such as α-glucosidase) can decrease the risk of complications such as cardiovascular disease, neuropathy, nephropathy, and angiopathy [1,2]. Inhibition of α-glucosidase can slow down the digestion of complex carbohydrates and, thus, reduce the release of glucose into the blood [3]. The clinically used α-glucosidase inhibitors (acarbose, voglibose, and miglitol) have common side effects, such as diarrhea and flatulence, with corresponding liver dysfunction and abdominal pain [4,5]. Thus, the search for new possible α-glucosidase inhibitors with few side effects is an important goal.

Plant-derived drugs contain natural compounds of various structures and are promising sources of α-glucosidase inhibitors [6,7]. Previously, we screened the most common tea species of the Rosaceae family growing in Siberia. High inhibitory activity of α-glucosidase (IC_50_ < 50 µg/mL) was a selection criterion and was used to identify promising plant species. Herb extracts of *Geum aleppicum* Jacq. and *Sibbaldianthe bifurca* (L.) Kurtto & T.Erikss. were the most active inhibitors of α-glucosidase according to their results [8]. *G. aleppicum* (*Colurieae* tribe) and *S. bifurca* (*Potentilleae* tribe) are closely related and belong to the Rosoideae subfamily [9].

*G. aleppicum* is an herbaceous perennial plant up to 70 cm high with an upright, reddish stem covered with stiff hairs. Basal leaves are long-petiolate and pinnate, with 3–6 pairs of cuneate–obovate lateral leaflets; the upper ones are trifoliate with large stipules and are rarely pubescent. The flowers are numerous, bright yellow, measuring 17–22 mm in diameter, rounded, on thick pedicels, and pubescent with short hairs. The native range of this species is the temperate Northern hemisphere. It grows in forests and steppe meadows, along forest edges, and near roads and residential areas [10]. The Buryat emchi-lamas use the *G. allepicum* herb decoction to treat diarrhea and indigestion [11]. Additionally, traditional Yakut medicine has long employed the *G. allepicum* decoction as an antidiabetic remedy [12]. *S. bifurca* (some scholars named it *Potentilla bifurca*) is a low shrub with woody stems in the lower part, is up to 30 cm tall, and is covered with harsh hairs. The stem leaves have oblong stipules and 2–7 pairs of lateral oblong leaflets that are obtuse at the apex. Flowers are bright yellow and measure 8–15 mm in diameter, with few-flowered apical inflorescence. *S. bifurca* grows in Siberian and Mongolian steppes and is found on the sandy coasts of North China [10]. This plant species is used in the Tibetan traditional medicine to treat diabetes [13]. Additionally, the extract from the whole plant is applied as an antitumor and antiulcerogenic remedy in Chinese traditional medicine [14]. 

The chemical composition of *G. aleppicum* has been insufficiently studied. The presence of some phenolic compounds, such as tiliroside, praecoxin D, eugenol, chlorogenic, gallic, salicylic acids, ethyl gallate [15], gemin A, and pedunculagin [16], is known. Additionally, triterpenoids daucosterol, β-sitosterol, and ursolic acid were previously identified [17]. Knowledge of the chemical composition of *S. bifurca* is low. Flavonoids have also been discovered including quercetin-4′-*O*-glucoside (spiraeoside) [18], quercetin-3-*O*-glucopyranoside, quercetin-3-*O*-xyloside, quercetin-3-*O*-(6″-*O*-*trans*-*p*-coumaroyl)-glucoside, quercetin, and myricetin [19]. Thus, there is a need for in-depth study of the chemical composition of these plant species. 

As part of an ongoing study on the metabolome of plant species of the Rosaceae family and their antidiabetic metabolites [8,20,21,22,23,24], we performed qualitative and quantitative chromatographic analyses of the chemical compounds for the first time in herbs of *G. aleppicum* and *S. bifurca* using high-performance liquid chromatography with photodiode array detection and electrospray ionization triple quadrupole mass spectrometric detection (HPLC-PDA-ESI-tQ-MS/MS). Additionally, herb extracts of *G. aleppicum* and *S. bifurca* were bioassayed using HPLC activity-based profiling to track metabolites with the highest α-glucosidase inhibitory potential.

## 2. Materials and Methods

### 2.1. Plant Material

Plant samples of *Geum aleppicum* herb were collected in the Republic of Buryatia, Kyakhtinsky District in 2022. The samples were collected in steppe meadow in eight locations, 10–12 samples from each (50°20′57.2338″ N, 106°25′37.1764″ E, 902 m a.s.l.). To identify patterns in the chemical composition, samples of *G. aleppicum* herb were harvested during various vegetation periods: active growth (23 May), flowering (15 July) and fruiting phases (10 September). Plant samples of *Sibbaldianthe bifurca* herb were collected in the Republic of Buryatia, Kyakhtinsky District in 2022. The samples were collected on the steppe area near the edge of the forest in 8 locations, with a total of 10–12 samples from each (50°21′13.0499″ N, 106°24′58.3707″ E. 880 m a.s.l.). To identify patterns in the chemical composition, samples of *S. bifurca* herb were harvested during various vegetation periods: active growth (16 May), flowering (8 July) and fruiting phases (12 September). The species were authenticated by Prof. Tamara A. Aseeva (IGEB SB RAS, Ulan-Ude, Russia). Experimental samples of herb were collected in the morning (between 9 and 11 h). The herb samples were sealed in plastic bags and placed in a cooler with ice for transport to the laboratory. The collected herb samples were dried in a ventilated hood at a temperature of 24 °C to a moisture content of 7–9%. The herb samples were stored at 4 °C before analysis in a Plant Repository of the Institute of General and Experimental Biology. To obtain herb samples with different growth periods, herbs from each collection date were pooled. After combining the herb samples from each collection date, three total samples of each growth period (active growth, May; flowering, July; fruiting, September) were obtained for both plants. No. GAL/Pro0522/12 (total sample, May), GAL/Pro0722/10 (total sample, July), GAL/Pro0922/12 (total sample, September) were the numbers of voucher specimens of *G. aleppicum* herb in the Plant Repository. No. SBI/Ros0522/10 (total sample, May), SBI/Ros0722/12 (total sample, July), SBI/Ros0922/12 (total sample, September) were the numbers of voucher specimens of *S. bifurca* herb in the Plant Repository. The samples were ground before analysis in an A11 basic analytical mill (IKA^®^-WerkeGmbh & Co. KG, Staufen, Germany). Hereinafter, herb samples were sieved up to an average particle diameter of 0.5 mm using sieving machine ERL-M1 (Zernotekhnika, Moscow, Russia).

### 2.2. Chemicals

The reference compounds were acquired from BenchChem (Austin, TX, USA): casuarictin (Cat. No. B1680760, ≥98%), casuariin (Cat. No. B1255675, ≥98%), casuarinin (Cat. No. B1208647, ≥98%); ChemFaces (Wuhan, China): *p*-hydroxybenzoic acid *O*-glucoside (Cat. No. CFN96590, ≥98%), niga-ichigoside F1 (Cat. No. CFN91060, ≥98%), rosamultin (Cat. No. CFN89097, ≥98%); Sigma–Aldrich (St. Louis, MO, USA): acetonitrile for HPLC (Cat. No. 34851, ≥99.9%), bovine serum albumin (Cat. No. A7030, ≥98%), citric acid (Cat. No. 251275, ≥99.5%), corosolic acid (Cat. No. PHL80065, ≥90%), 3,4-dihydroxybenzoic acid 4-*O*-glucoside (Cat. No. E24859, ≥97%), ellagic acid (Cat. No. PHL89653, ≥98%), gallic acid (Cat. No. 398225, ≥98%), glucose (Cat. No. G8270, ≥99.5%), α-glucosidase from *Saccharomyces cerevisiae* (G5003), Cat. No. kaempferol-3-*O*-glucoside (astragalin; Cat. No. 68437, ≥90%), kaempferol-3-*O*-glucuronide (Cat. No. 79273, ≥97%), lithium perchlorate (Cat. No. 205281, ≥95%), malic acid (Cat. No. PHR1273, ≥99.5%), *p*-nitrophenyl-α-d-glucopyranoside (Cat. No. 487506), perchloric acid (Cat. No. 244252, ≥70%), quercetin-3-*O*-arabinoside (Cat. No. 75759, ≥95%), quercetin-3-*O*-glucoside (isoquercitrin; Cat. No. 16654, ≥98%), quercetin-3-*O*-glucuronide (Cat. No. 90733, ≥90%), sucrose (Cat. No. S0389, ≥99.5), 3,4,5-trihydroxybenzaldehyde (Cat. No. 259594, ≥98%), tormentic acid (Cat. No. PHL85836, ≥95%), ursolic acid (Cat. No. U6753, ≥90%); Toronto Research Chemicals (North York, Toronto, ON, Canada): pedunculagin (Cat. No. P354070, ≥95%). 2-Pyrone-4,6-dicarboxylic acid, agrimoniin and potentillin were previously isolated from *Comarum palustre* herb [22]; tellimagrandins, rugosins were isolated earlier from *Filipendula ulmaria* herb [23]; gemin A was isolated from *Potentilla anserina* herb [24]; 1-*O*-*p*-hydroxybenzoic acid *O*-glucoside was isolated from *Calendula officinalis* leaves [25]; quercetin-3-*O*-(6″-*O*-cinnamoyl)-glucoside was previously isolated from *Rhaponticum uniflorum* leaves [26]. 

### 2.3. Plant Extracts Preparation

To prepare plant extracts, 10 g of dry and grounded herb of *G. aleppicum* and *S. bifurca* were extracted twice via stirring in a glass flask (200 mL) with 70% methanol (100 mL) with sonication at 40 °C using a Sapphire 2.8 bath (Sapphire Ltd., Moscow, Russia) for 30 min, ultrasound power 100 W, and frequency 35 kHz. The obtained methanolic extracts were combined, filtered through a cellulose filter and concentrated under reduced pressure until dryness. Obtained extracts were stored at 4 °C before use for HPLC analysis and α-glucosidase inhibiting activity study. The yields of total extracts of *G. aleppicum* were 3.3 g (May sample), 3.5 g (July sample), 3.2 g (September sample). The yields of total extracts of *S. bifurca* were 2.8 g (May sample), 3.2 g (July sample), 3.0 g (September sample). Before analysis, dry extract (100 mg) was dissolved in 10 mL 70% methanol using measuring flask (10 mL) and filtered through 0.22 μm syringe filters. 

### 2.4. High-Performance Liquid Chromatography with Photodiode Array Detection and Electrospray Ionization Triple Quadrupole Mass Spectrometric Detection (HPLC-PDA-ESI-tQ-MS/MS) Metabolite Profiling

To analyze the chemical profile of *G. aleppicum* and *S. bifurca* herb extracts, the previously described method used high-performance liquid chromatography with photodiode array detection and electrospray ionization triple quadrupole mass spectrometric detection (HPLC-PDA-ESI-tQ-MS/MS) was applied [8]. Chromatographic separation of compounds was realized with liquid chromatograph LC-20 Prominence coupled with a photodiode array detector, SPD-M30A (wavelength range of 200–600 nm), and a triple-quadrupole mass spectrometer, LCMS 8050 (all Shimadzu, Columbia, MD, USA). Column GLC Mastro C18 (2.1 × 150 mm, 3 μm) was used. Column temperature was 30 °C. The following eluents were used: A (0.4% formic acid in water) and B (0.4% formic acid in acetonitrile). The injection volume was 1 μL, and elution flow rate was 80 μL/min. Gradient program: 0.0–2.0 min 5.0–7.5% B, 2.0–7.0 min 7.5–15.0% B, 7.0–11.0 min 15.0–38.0% B, 11.0–14.0 min 38.0–42.0% B, 14.0–20.0 min 42.0–80.0% B, 20.0–25.0 min 80.0–100.0% B, 25.0–35.0 min 100.0–5.0% B. The negative electrospray ionization was applied for mass spectrometric detection (–3 kV source voltage, range of *m*/*z* 100–1900, collision energy 5–40 eV). There were following temperature levels of ESI interface (300 °C), desolvation line (250 °C), and heat block (400 °C). There were following flow rates of nebulizing gas (N_2_, 3 L/min), heating gas (air, 10 L/min), collision-induced dissociation gas (Ar, 0.3 mL/min). The data were processed with LabSolution’s workstation software (Shimadzu) equipped with the inner LC-MS library. The identification of metabolites was realized via the analysis of their retention time, ultraviolet, and mass-spectrometric data comparing the same criteria with the reference standards and literature data. 

### 2.5. HPLC-PDA-ESI-tQ-MS/MS Metabolite Quantification

For the quantification of 34 compounds of *G. aleppicum* and *S. bifurca* herb extracts in known HPLC-PDA-ESI-tQ-MS/MS conditions (Section 2.4), the following reference compounds were used: saccharose, glucose, malic acid, citric acid, 2-pyrone-4,6-dicarboxylic acid, gallic acid, 3,4-dihydroxybenzoic acid 4-*O*-glucoside, 3,4,5-trihydroxybenzaldehyde, pedunculagin, 1-*O*-*p*-hydroxybenzoic acid *O*-glucoside, casuariin, tellimagrandin I_1_, tellimagrandin I_2_, rugosin E_1_, casuarinin, rugosin E_2_, potentillin, casuarictin, agrimoniin, gemin A, tellimagrandin II_2_, quercetin-3-*O*-glucuronide, quercetin-3-*O*-glucoside, quercetin-7-*O*-glucoside, quercetin-3-*O*-arabinoside, quercetin-3-*O*-(6″-*O*-cinnamoyl)-glucoside, ellagic acid, kaempferol-3-*O*-glucoside, kaempferol-3-*O*-glucuronide, niga-ichigoside F1, rosamultin, tormentic acid, corosolic acid and ursolic acid. For the preparation of stock solutions (1000 µg/mL), 10 mg of reference compounds were separately weighted and dissolved in the methanol-DMSO mixture (1:1) in volumetric flasks (10 mL) followed by the creation of ‘concentration–peak area’ graphs (1–100 µg/mL). The values of correlation coefficient (r^2^), standard deviation (S_YX_), limit of detection (LOD), limit of quantification (LOQ), and linear range were calculated in Advanced Grapher 2.2 (Alentum Software Inc., Ramat-Gan, Israel) using calibration curve data [27] and the results of three sufficient HPLC runs (Table S1). The parameters of intra-day, inter-day precisions and recovery of spiked sample were investigated using the known method [28]. The obtained results were presented as mean values ± standard deviation (S.D.).

### 2.6. HPLC Activity-Based Profiling

To perform HPLC activity-based profiling, aliquots (100 µL) of *G. aleppicum* herb extract solution (10 mg/mL) and *S. bifurca* herb extract solution (10 mg/mL) were separated under analytical HPLC-PDA-ESI-tQ-MS/MS conditions as described in Section 2.4. The collection of eluates (40 µL) was performed every 30 s in 96-well plates. Then, the eluates were dried and redissolved in 10 µL of phosphate-buffered saline (PBS) followed by analysis as described previously [20]. α-Glucosidase from *Saccharomyces cerevisiae* was dissolved in PBS (pH 6.8), which contained bovine serum albumin (0.2%) up to 0.5 U/mL concentration, then 125 µL of PBS and 60 µL *p*-nitrophenyl-α-d-glucopyranoside (5 mM) were added. The incubation of the samples was realized at 37 °C for 5 min. Thereafter, 60 μL of α-glucosidase (0.4 U/mL) was added. Then, the samples were incubated at 37 °C for 15 min and 50 µL of sodium carbonate (200 mM) was added. Absorbance was determined at 400 nm. Epicatechin gallate was the reference compound. The activity of the microfractions as a percentage from the activity of the reference compound was displayed on the chromatogram as bars.

### 2.7. Statistical Analysis

Statistical analyses were performed using one-way analysis of variance, and the significance of the mean difference was determined using Duncan’s multiple range test. Differences at *p* < 0.05 were considered statistically significant. The results are presented as the mean ± S.D. The linear regression analysis and generation of calibration graphs were conducted using Advanced Grapher 2.2 (Alentum Software, Inc., Ramat-Gan, Israel).

## 3. Results and Discussion 

### 3.1. Metabolites of Geum aleppicum Herb: HPLC-PDA-ESI-tQ-MS/MS Profile

**Figure 1:**
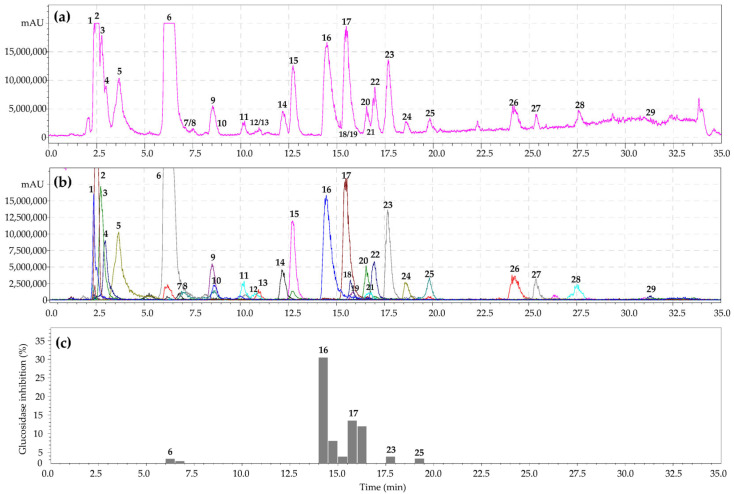
High-performance liquid chromatography with electrospray ionization triple quadrupole mass spectrometric detection (hPlC-PDA-ESI-tQ-MS/MS) base peak chromatogram (**a**); selected ion monitoring mode (SIM), negative ionization (**b**); HPLC activity-based profiling of α-glucosidase-inhibition (**c**) of *G. aleppicum* herb extract (July sample). Compounds are numbered as listed in Table 1.

The *Geum* genus is characterized by the presence of numerous chemical classes of compounds with definite chromatographic behavior [29]. High-performance liquid chromatography with photodiode array and electrospray ionization triple quadrupole mass spectrometric detection (HPLC-PDA-ESI-tQ-MS/MS) was applied to separate compounds from the *G. aleppicum* herb extract. Analysis of chromatographic mobility, UV parameters, and mass spectral data and subsequent comparison of the obtained results with reference standards and/or literature information led to the identification of 29 compounds of various chemical classes (Figure 1a,b; Table 1).

#### 3.1.1. Carbohydrates

Two carbohydrates were discovered in *G. aleppicum* herb extract including saccharose (**1**) and glucose (**2**). Earlier, glucose was revealed in the herb and roots of *G. urbanum* and leaves of *G. montanum* [30]. Additionally, saccharose was detected in the herbs of *G. montanum* [30] and *G. rivale* [31] and roots of *G. iranicum* [32]. 

#### 3.1.2. Organic Acids 

The presence of malic (**3**) and citric (**4**) acids was noted for the *G. aleppicum* herb. Previously, malic acid was found in the aerial parts of *G. reptans*, *G. montanum*, *G. bulgaricum*, and *G. hybrid* [33]. There are no data on the detection of citric acid in other species of the genus *Geum.*

#### 3.1.3. Benzoic Acid Derivatives 

Three benzoic acid derivatives were determined in the *G. aleppicum* herb. 3,4-Dihydroxybenzoic acid 4-*O*-glucoside (**6**) and 3,4,5-trihydroxybenzaldehyde (**7**) were identified by comparing these with reference standards. The mass spectrometric analysis of compound **9** demonstrated the loss of a hexose fragment (162 Da) and the remaining fragment with *m*/*z* 121 corresponding to a benzoic acid moiety. The assumed structure of compound **9** was found to be a benzoic acid, *O*-hexoside. 3,4,5-Trihydroxybenzaldehyde was revealed earlier in *G. japonicum* [34], while 3,4-dihydroxybenzoic acid 4-*O*-glucoside was found in *Geum* for the first time.

#### 3.1.4. Ellagic Acid Derivatives and Ellagitannins

Ellagic acid (**19**), two ellagic acid glycosides (**11**, **12**), ellagic acid ether (**27**), and three ellagitannins (**8**, **10**, **16**) were detected in the *G. aleppicum* herb. The presence of ellagitannins in *G. aleppicum* confirms the regularity of their presence in the Rosaceae family as a chemotaxonomic marker [16]. Comparison with reference standards allowed the identification of ellagic acid (**19**) and ellagitannins of different structural types according to the classification by Okuda et al. [35] such as hexahydroxyphenoyl glucose (pedunculagin, **8**), *C*-glycosidic (casuariin, **10**), and dehydrodigalloyl (gemin A, **16**). The mass spectrometric analysis of **11** and **12** showed the loss of a pentosyl moiety and a methyl fragment (14 Da), leaving the moiety with *m*/*z* 301, which is specific for ellagic acid derivatives. The provisional structures of **11** and **12** were found to be the ellagic acid methyl ether *O*-pentosides. Previously, ellagic acid was detected in the *G. rivale* aerial part [36], *G. urbanum* rhizome [37], and *G. japonicum* whole plants [38]. Earlier, gemin A was identified in *G. urbanum* roots [39], *G. japonicum* leaves [40], *G. rivale* leaves [41], and *G. aleppicum* leaves [16]. Pedunculagin was revealed in *G. aleppicum* leaves [16], *G. urbanum* roots [29], and the leaves of *G. aleppicum* and *G. calthifolium* [16]. Casuariin was identified in the whole plant of *G. japonicum* [42] and *G. urbanum* roots [39]. Thus, ellagic acid and casuariin were detected in *G. aleppicum* for the first time. 

#### 3.1.5. Flavonoids 

Six flavonoids were determined in the *G. aleppicum* herb extract as flavonols in the glycoside state. Depending on the flavonol structure of aglycone, they belonged to the quercetin (**13**, **17**, **18**) or kaempferol (**14**, **20**, **21**) groups. Quercetin-3-*O*-glucuronide (**17**), quercetin-3-*O*-glucoside (**18**), kaempferol-3-*O*-glucuronide (**20**), and kaempferol-3-*O*-glucoside (**21**) were successfully identified in the *G. aleppicum* herb using reference standards. Compound **13** was the acidic derivative of quercetin and gave the characteristic fragments of *m*/*z* 477 (quercetin *O*-hexuronide) and *m*/*z* 301 (quercetin). The MS pattern of compound **14** showed the loss of the fragment *m*/*z* 176 (which is characteristic of hexuronic acid) and the presence of a moiety with *m*/*z* 285 (corresponding to kaempferol). The provisional structures of compounds **13** and **14** were quercetin-*O*-hexuronide-*O*-hexuronide and kaempferol-*O*-hexuronide-*O*-hexuronide, respectively. 

Previously, the presence of quercetin-3-*O*-glucoside was shown in the aerial part of *G. rivale* [36] and *G. bulgaricum* [43]; quercetin-3-*O*-glucuronide was revealed in the aerial part of *G. rivale* [36] and leaves of *G. calthifolium* var. *nipponicum* [44]. Additionally, kaempferol-3-*O*-glucoside was detected in the aerial part of *G. rivale* [36], *G. bulgaricum* [43], the whole plant of *G. japonicum* [45], and the herb of *G. urbanum* [46], while kaempferol-3-*O*-glucuronide was found in the aerial part of *G. rivale* [36] and leaves of *G. calthifolium* var. *nipponicum* [44]. Thus, the presence of quercetin-3-*O*-glucuronide, quercetin-3-*O*-glucoside, kaempferol-3-*O*-glucuronide, and kaempferol-3-*O*-glucoside was revealed in the *G. aleppicum* herb for the first time. 

#### 3.1.6. Triterpenoids 

Six triterpenoids were identified in the *G. aleppicum* herb including niga-ichigoside F1 (**23**) and its isomer (**22**), rosamultin (tormentic acid *O*-glucoside, **25**), and tormentic (**26**), corosolic (**28**), and ursolic (**29**) acids. All of the revealed compounds were ursane-type triterpenoids, which are often found in species of the *Geum* genus [15]. Previously, only ursolic acid was found in *G. aleppicum* [17]. Earlier, niga-ichigoside F1 was determined in the *G. japonicum* plant [47], the aerial part of *G. rivale* [36], the roots of *G. urbanum* [48], and in the *G. japonicum* Thunb. var. *chinense* plant [15]. Rosamultin was found in the *G. japonicum* plant [49]. Tormentic acid was found in the aerial part of *G. rivale* [36], the *G. japonicum* plant [47], roots of *G. urbanum* [48], and the *G. japonicum* Thunb. var. *chinense* plant [15]. Corosolic acid was found in the *G. japonicum* plant [49]; ursolic acid was found in the aerial part of *G. rivale* [36], whole plant of *G. japonicum* [50], and *G. urbanum* roots [51]. Thus, niga-ichigoside F1 and rosamultin, tormentic, and corosolic acids were discovered in the *G. aleppicum* herb for the first time.

### 3.2. Metabolites of Sibbaldianthe bifurca Herb: HPLC-PDA-ESI-tQ-MS/MS Profile

**Figure 2:**
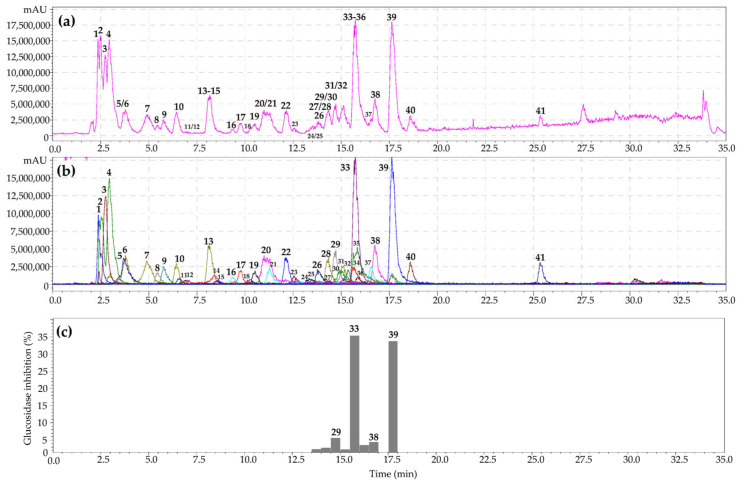
High-performance liquid chromatography with electrospray ionization triple quadrupole mass spectrometric detection (HPLC-PDA-ESI-tQ-MS/MS) base peak chromatogram (**a**); selected ion monitoring mode (SIM), negative ionization (**b**); HPLC activity-based profiling of α-glucosidase-inhibition (**c**) of *S. bifurca* herb extract (July sample). Compounds are numbered as listed in Table 2.

HPLC–PDA–ESI–tQ–MS/MS was used to separate metabolites from the *S. bifurca* herb extract. Analysis of chromatographic mobility, UV parameters, and mass spectral data and subsequent comparison of the obtained results with reference standards and/or literature information led to the identification of 41 compounds of various chemical classes (Figure 2a,b; Table 2).

#### 3.2.1. Carbohydrates and Organic Acids 

The carbohydrates [saccharose (**1**) and glucose (**2**)] and two organic acids [malic (**3**) and citric (**4**)] were discovered in the *S. bifurca* herb upon comparison of *t*_r_, UV, and mass spectra data with reference standards. Previously, these compounds were not detected in the *Sibbaldianthe* genus. 

#### 3.2.2. Galloyl O-Glycosides 

Gallic acid (**8**) in the free state and twelve of its hexosides (**3**, **6**, **7**, **11**, **14**, **17**–**20**, **22**, **29**, **34**) were detected in the *S. bifurca* herb extract. Found hexosides were characterized by the number of galloyls: one (monogalloyl hexose, **3**, **6**, **7**), two (digalloyl hexose, **11**, **14**, **17**), three (trigalloyl hexose, **18**–**20**, **22**), four (tetragalloyl hexose, **29**), and five (pentagalloyl hexose, **34**). Gallic acid was detected using the reference standard, while galloyl *O*-hexosides were identified in the mass spectrum with deprotonated ions [M–H]^−^ of *m*/*z* 331 (mono-), 483 (di-), 635 (tri-), 787 (tetra-), and 939 (penta-), and daughter ions related to the loss of gallic acid. Gallic acid was not previously found in the *Sibbaldianthe* genus.

#### 3.2.3. Benzoic Acid Derivatives

Three benzoic acid derivatives (**9**, **10**, and **13**) were determined in the *S. bifurca* herb. 3,4-Dihydroxybenzoic acid 4-*O*-glucoside (**9**) and 1-*O*-*p*-hydroxybenzoic acid *O*-glucoside (**13**) were identified by comparing these with reference standards. Compound **10** was established as *p*-hydroxybenzoic acid *O*-hexoside owing to specific UV (274 nm) and mass spectral patterns with the loss of a hexose fragment (162 Da) and the presence of ions with *m*/*z* 137 corresponding to the hydroxybenzoic acid moiety. 3,4-Dihydroxybenzoic acid 4-*O*-glucoside and 1-*O*-*p*-hydroxybenzoic acid *O*-glucoside were found in the *Sibbaldianthe* genus for the first time.

#### 3.2.4. Ellagic Acid Derivatives and Ellagitannins

Ellagic acid (**35**), ellagic acid ether (**41**), and thirteen ellagitannins (**12**, **15**, **16**, **21**, **23**–**28**, **30**, **31**, **36**) were revealed in the *S. bifurca* herb. The identification of ellagic acid (**35**) and ellagitannins of different structural types, such as hexahydroxyphenoyl glucose (pedunculagin, **12**), *C*-glycosidic (casuariin, **10**; casuarinin, **25**; casuarictin, **28**), dehydrodigalloyl (agrimoniin, **30**), hexahydroxyphenoylgalloyl glucose (potentillin, **27**; tellimagrandins: I_1_, **16**; I_2_, **21**; II_2_, **31**), and valoneoyl (rugosin E_1_, **24**; rugosin E_2_, **26**), was realized via comparison with reference standards [52]. The mass spectra of **23** and **36** gave typical ions of the deprotonated molecules [M–H]^−^ (*m*/*z* 951 and 1087, respectively) and double-charged molecules [M–2H]^2−^ (*m*/*z* 475 and 543, respectively). Provisional structures of **23** and **26** were found to be trigalloyl-hexahydroxydiphenoyl-hexoside and digalloyl-bis-hexahydroxydiphenoyl-hexoside, respectively [21,53]. The mass spectra of **41** showed the loss of a methyl fragment (14 Da) and the presence of a fragment with *m*/*z* 301, which is specific to ellagic acid. The provisional structure of **41** was found to be an ellagic acid methyl ether. Previously, ellagitannins had not been found in the genus *Sibbaldianthe*.

#### 3.2.5. Flavonoids

The flavonoid profile of the *S. bifurca* herb was similar to that of the *G. aleppicum* herb, i.e., the presence of quercetin (**33**, **38**–**40**) and kaempferol (**32**, **37**) derivatives was found. All six flavonoids were revealed in the glycoside state. Quercetin-3-*O*-glucuronide (**33**), kaempferol-3-*O*-glucuronide (**37**), quercetin-7-*O*-glucoside (**38**), quercetin-3-*O*-arabinoside (**39**), and quercetin-3-*O*-(6″-*O*-cinnamoyl)-glucoside were identified in the *S. bifurca* herb upon comparison of *t*_r_, UV, and mass spectral data to reference standards. Compound **32** gave the deprotonated ion [M–H]^−^ with *m*/*z* 609 and the aglycone fragment in the MS^2^ spectra at *m*/*z* 285, which is characteristic for kaempferol. Additionally, the loss of a hexosyl moiety was observed. The tentative structure of **32** was kaempferol-*O*-hexoside-hexoside. Quercetin-3-*O*-glucuronide, kaempferol-3-*O*-glucuronide, quercetin-7-*O*-glucoside, quercetin-3-*O*-arabinoside, and quercetin-3-*O*-(6″-*O*-cinnamoyl)-glucoside were found in the *Sibbaldianthe* genus for the first time.

### 3.3. Quantitative Content and Seasonal Variation of Profile of Geum aleppicum and Sibbaldianthe bifurca Herb

To identify possible patterns in the chemical profile of the *G. aleppicum* herb and *S. bifurca* herb, these species were collected and investigated at different growth phases: active growth (May), flowering (July), and fruiting (September). The maximum content of the majority of compounds in both species was observed during the flowering period. In particular, gemin A, miquelianin (quercetin-3-*O*-glucuronide), niga-ichigoside F1, 3,4-dihydroxybenzoic acid 4-*O*-glucoside, and glucose were the dominant compounds of the *G. aleppicum* herb. The contents of different ellagitannins increased towards the flowering phase and then decreased upon fruiting. Thus, the content of the dominant ellagitannin, gemin A, in the active growth phase (10.18 mg/g) increased more than five times by the flowering phase (53.26 mg/g), and then, it gradually decreased in the fruiting stage (42.11 mg/g). A similar trend was observed for both casuariin (1.26 mg/g → 2.57 mg/g → 2.03 mg/g) and pedunculagin (trace → 0.26 mg/g → trace). In contrast, the content of ellagic acid was the maximum in the fruiting phase (5.63 mg/g), which occurred possibly because ellagic acid was released during the hydrolysis of ellagitannins [54]. Flavonoids, both derivatives of quercetin and kaempferol, accumulated the most in the flowering phase of the *G. aleppicum* herb. The content of the prevalent quercetin derivative, miquelianin, in the active growth phase increased from 5.20 mg/g to 26.83 mg/g in the flowering period. One possible reason for the maximum accumulation of flavonoids in the *G. aleppicum* herb in the flowering phase may be the high UV radiation and air temperature. Previously, similar accumulations of flavonols at high growth temperatures were observed in other representatives of the Rosaceae family [21,55].

Guaiaverin (quercetin-3-*O*-arabinoside), miquelianin, tellimagrandin II_2_, casuarictin, and glucose were the dominant compounds in the *S. bifurca* herb. The accumulation of the dominant flavonoids, guaiaverin and miquelianin, was also observed during the growth phase (21.59 and 19.62 mg/g, respectively). The concentrations of dominant ellagitannins, tellimagrandin II_2_ and casuarictin, increased until the flowering stage and then consistently decreased until the fruiting period (3.62 mg/g → 7.83 mg/g → 5.16 mg/g and 1.60 mg/g → 5.28 mg/g → 4.16 mg/g, respectively). The maximum content of gallotannins was observed in samples during the growth phase, followed by a decrease in the flowering and fruiting phase samples. This can likely be explained by the fact that galloyl hexoses are precursors of complex hydrolysable tannins, the biosynthesis of which is carried out via oxidative binding of galloyl groups [56,57]. Thus, the maximum accumulation of dominant ellagitannins and flavonoids in the *G. aleppicum* and *S. bifurca* herbs under Siberian conditions was observed during the flowering phase in July.

### 3.4. Chemotaxonomic Significance of G. aleppicum and S. bifurca Metabolites

As a result of the chromatographic investigation of the *G. allepicum* and *S. bifurca* herbs, 70 metabolites of different chemical classes were identified. To select compounds of chemotaxonomic significance for these species, particular attention should be paid to 2-pyrone-4,6-dicarboxylic acid, hydrolysable ellagitannins, and flavonols.

Currently, *S. bifurca* belongs to the Potentilleae tribe [58], and *G. aleppicum* belongs to the Colluria tribe, although earlier, experts attributed it to the tribe Dryadeae [9]. Both species are closely related and belong to the Rosoideae subfamily [9]. 2-Pyrone-4,6-dicarboxylic acid is a breakdown product of phenolic compounds and is a taxonomic marker of the Rosoideae subfamily [59]. Both the *G. aleppicum* and *S. bifurca* herbs contained 2-pyrone-4,6-dicarboxylic acid, which confirmed the results reported by Wilkes et al. on the presence of this compound in representatives of the Rosoideae subfamily [59].

Ellagitannins have a wide distribution in the Rosaceae family [60], while oligomeric hydrolysable tannins are limited to the Rosoideae subfamily [16]. According to this theory, most species of the Rosoideae subfamily contain one or two oligomers that are used as chemotaxonomic markers. In the studied plant objects, dimer gemin A for the *G. aleppicum* herb and dimers rugosin E and agrimoniin for the *S. bifurca* herb may have chemotaxonomic significance.

Flavonoids have also been proposed as a chemotaxonomic marker of the Rosaceae family [61]. Derivatives of kaempferol and quercetin were revealed in both the *G. aleppicum* and *S. bifurca* herbs. However, these species did not contain any specific flavonoids that would allow us to discuss chemosystematic markers. Thus, the exact chemosystematic significance of flavonols in the Rosoideae subfamily is not definitively due to their wide presence in Rosaceae in general.

### 3.5. α-Glucosidase Inhibiting Activity of Geum aleppicum and Sibbaldianthe bifurca Herb Extract: HPLC Activity-Based Profiling

To reveal the components of *G. aleppicum* and *S. bifurca* herb extracts with α-glucosidase-inhibiting properties, the HPLC activity-based profiling method was used. This is a highly multipurpose strategy to miniaturize and accelerate identification of active substances in analyzed extracts by analytical HPLC [62,63]. HPLC activity-based profiling is performed via post-column collection of microfractions in a plate after a certain period of time, their subsequent drying, and the addition of reagents for biological evaluation [64,65]. The activity of the microfractions as a percentage of the activity of the reference compound is displayed on the chromatogram as bars (Figure 1c). Epicatechin gallate was chosen as the reference compound due to its high ability to inhibit α-glucosidase with IC_50_ value of 4.03 ± 0.01 μg/mL [66]. Epicatechin gallate isolated from *Rhodiola crenulata* roots inhibited α-glucosidase with IC_50_ 0.71 ± 0.01 [67].

As a result of HPLC activity-based profiling of the *G. aleppicum* herb extract, inhibition of α-glucosidase by 3,4-dihydroxybenzoic acid 4-*O*-glucoside, gemin A, quercetin-3-*O*-glucuronide, niga-ichigoside F1, and rosamultin was found. The most pronounced inhibition of α-glucosidase was observed for ellagitannin gemin A and flavonol quercetin-3-*O*-glucuronide. The same procedure for the *S. bifurca* herb extract revealed inhibition of α-glucosidase by tetragalloyl hexose, quercetin 3-*O*-glucuronide, quercetin-7-*O*-glucoside, and quercetin-3-*O*-arabinoside (Figure 2c). Maximal inhibition of α-glucosidase was found for the flavonols quercetin-3-*O*-glucuronide and quercetin-3-*O*-arabinoside. Previously, it was suggested that the mechanism of the inhibitory activity of ellagitannins against α-glucosidase involves their binding to proteins, followed by changes in the conformation of the enzyme and a decrease in its activity [68,69]. In turn, flavonols bind to glucosidase with high affinity through hydrogen bonds and van der Waals forces, and then, these complexes lead to conformational changes in α-glucosidase [70,71]. Thus, microfractionation of the *G. aleppicum* and *S. bifurca* herb extracts allowed direct evaluation of the α-glucosidase inhibitory activity of the components in their biological matrices. The obtained results can serve as a basis for using these plant compounds as possible sources for hypoglycemic nutraceutical production.

## 4. Conclusions

The *Geum aleppicum* and *Sibbaldianthe bifurca* herbs are used in traditional medicine as antidiabetic remedies. In an attempt to identify compounds with antidiabetic potential, these closely related plant species were first characterized via HPLC-PDA-ESI-tQ-MS/MS and, as a result, data on 70 compounds were obtained. Carbohydrates, organic acids, derivatives of benzoic and ellagic acids, ellagitannins, flavonoids and triterpenoids were identified in both plant species. Then, HPLC activity-based profiling was applied, which allowed one to miniaturize and accelerate the identification of active substances in the analyzed extracts. Gemin A, quercetin-3-*O*-glucuronide and quercetin-3-*O*-arabinoside showed the most pronounced results in terms of α-glucosidase inhibition. In this study, the *G. aleppicum* and *S. bifurca* herbs were shown to be natural sources of metabolites with α-glucosidase-inhibiting properties. Further in vivo investigations of these plant extracts are necessary for the wide introduction of new biologically active agents into therapeutic practice for the treatment of diabetes mellitus.

## Figures and Tables

**Table 1:** Chromatographic (*t*_r_), UV and mass-spectrometric data, and seasonal presence/content of compounds **1**–**29** found in *G. aleppicum* herb extract.

No	*t*_r_, min	Compound ^a^	UV, nm	[M–H]^−^, MS/MS, *m*/*z*	Seasonal Content, mg/g DW ± SD		
					May	**July**	**September**
**1**	2.39	Saccharose ^S^	205	341 [M–H]^−^	2.39 ± 0.13	24.18 ± 1.23	41.68 ± 2.55
**2**	2.55	Glucose ^S^	205	179 [M–H]^−^	3.86 ± 0.23	48.52 ± 2.96	27.11 ± 1.63
**3**	2.78	Malic acid ^S^	205	133 [M–H]^−^	2.63 ± 0.14	5.69 ± 0.36	9.18 ± 0.53
**4**	3.04	Citric acid ^S^	205	191 [M–H]^−^	0.93 ± 0.05	3.11 ± 0.20	5.73 ± 0.36
**5**	3.66	2-Pyrone-4,6-dicarboxylic acid ^S^	314	183 [M–H]^−^; [183]→139 [(M–H)–CO_2_]^−^, 111 [(M–H)–COOH–CO]^−^	0.83 ± 0.04	5.29 ± 0.32	5.33 ± 0.32
**6**	6.32	3,4-Dihydroxybenzoic acid 4-*O*-Glc ^S^	297	315 [M–H]^−^; [315]→153 [(M–H)–Glc]^−^	2.44 ± 0.17	11.27 ± 0.62	10.86 ± 0.68
**7**	6.72	3,4,5-Trihydroxybenzaldehyde ^S^	275	153 [M–H]^−^	traces	0.08 ± 0.01	traces
**8**	7.04	Pedunculagin ^S^	270	783 [M–H]^−^; 391 [M–2H]^2−^	traces	0.26 ± 0.02	traces
**9**	8.50	Benzoic acid *O*-Hex ^L^	265	283 [M–H]^−^; [283]→121 [(M–H)–Hex]^−^	0.83 ± 0.05	4.33 ± 0.28	4.08 ± 0.22
**10**	8.59	Casuariin ^S^	270	783 [M–H]^−^; 391 [M–2H]^2−^	1.26 ± 0.08	2.57 ± 0.15	2.03 ± 0.12
**11**	10.16	Ellagic acid methyl ether *O*-Pent ^L^	254, 360	447 [M–H]^−^; [447]→315 [(M–H)–Pent]^−^; [315]→301 [(M–H)–Pent–CH_2_]^−^	traces	0.54 ± 0.03	0.10 ± 0.01
**12**	10.82	Ellagic acid methyl ether *O*-Pent ^L^	254, 360	447 [M–H]^−^; [447]→315 [(M–H)–Pent]^−^; [315]→301 [(M–H)–Pent–CH_2_]^−^	traces	0.32 ± 0.02	traces
**13**	10.96	Quercetin-*O*-HexA-*O*-HexA ^L^	254, 267, 351	653 [M–H]^−^; [653]→477 [(M–H)–HexA]^−^, 301 [(M–H)–2×HexA]^−^	traces	0.82 ± 0.05	traces
**14**	12.29	Kaempferol-*O*-HexA-*O*-HexA ^L^	265, 345	637 [M–H]^−^; [637]→461 [(M–H)–HexA]^−^, 285 [(M–H)–2×HexA]^−^	0.26 ± 0.01	1.89 ± 0.13	0.29 ± 0.02
**15**	12.73	Feruloyl tartronic acid ^L^	296, 327	295 [M–H]^−^; [295]→193 [(M–H)–102]^−^	1.07 ± 0.07	3.97 ± 0.25	2.18 ± 0.13
**16**	14.47	Gemin A ^S^	270	1871 [M–H]^−^; 935 [M–2H]^2−^	10.18 ± 0.70	53.26 ± 3.25	42.11 ± 2.11
**17**	15.49	Quercetin-3-*O*-GlcA ^S^	254, 268, 352	477 [M–H]^−^; [477]→301 [(M–H)–GlcA]^−^	5.20 ± 0.30	26.83 ± 1.58	10.75 ± 0.59
**18**	15.84	Quercetin-3-*O*-Glc ^S^	254, 285, 355	463 [M–H]^−^; [463]→301 [(M–H)–Glc]^−^	traces	1.28 ± 0.07	traces
**19**	15.88	Ellagic acid ^S^	255, 367	301 [M–H]^−^	traces	0.89 ± 0.05	5.63 ± 0.38
**20**	16.55	Kaempferol-3-*O*-GlcA ^S^	265, 344	461 [M–H]^−^; [461]→285 [(M–H)–GlcA]^−^	1.03 ± 0.06	2.97 ± 0.18	2.08 ± 0.11
**21**	16.66	Kaempferol-3-*O*-Glc ^S^	265, 343	447 [M–H]^−^; [447]→285 [(M–H)–Glc]^−^	traces	0.52 ± 0.03	traces
**22**	16.92	Niga-ichigoside F1 isomer ^L^	210	665 [M–H]^−^; [665]→503 [(M–H)–Glc]^−^	1.22 ± 0.07	2.01 ± 0.12	2.09 ± 0.13
**23**	17.69	Niga-ichigoside F1 ^S^	210	665 [M–H]^−^; [665]→503 [(M–H)–Glc]^−^	4.27 ± 0.23	10.82 ± 0.66	8.12 ± 0.44
**24**	18.62	Gallocatechin gallate *O*-gallate ^L^	250	593 [M–H]^−^; [593]→441 [(M–H)–GallA]^−^	traces	2.93 ± 0.17	1.14 ± 0.06
**25**	19.84	Rosamultin (tormentic acid *O*-Glc) ^S^	210	649 [M–H]^−^; [649]→487 [(M–H)–Glc]^−^	0.74 ± 0.05	1.73 ± 0.10	1.93 ± 0.11
**26**	24.27	Tormentic acid ^S^	210	487 [M–H]^−^	0.52 ± 0.04	1.14 ± 0.07	1.37 ± 0.08
**27**	25.42	Ellagic acid methyl ether ^L^	254, 362	315 [M–H]^−^; [315]→301 [(M–H)–CH_2_]^−^	0.14 ± 0.01	0.63 ± 0.04	1.83 ± 0.11
**28**	27.50	Corosolic acid ^S^	210	471 [M–H]^−^	0.10 ± 0.01	0.43 ± 0.02	0.69 ± 0.04
**29**	31.31	Ursolic acid ^S^	210	455 [M–H]^−^	traces	0.05 ± 0.00	0.67 ± 0.04

^a^ Compound identification was based on comparison of retention time and MS spectral data with reference standard (^S^) or interpretation of MS spectral data and comparison with literature data (^L^). Traces—<LOQ (limit of quantification). Abbreviation used: Glc—glucose; Hex—hexose; Pent—pentose; HexA—hexuronic acid; GlcA—glucuronic acid.

**Table 2:** Chromatographic (*t*_r_), UV and mass-spectrometric data, and seasonal presence/content of compounds **1**–**41** found in *S. bifurca* herb extract.

No	*t*_r_, min	Compound ^a^	UV, nm	[M–H]^−^, MS/MS, *m*/*z*	Seasonal Content, mg/g DW ± SD		
					May	**July**	**September**
**1**	2.39	Saccharose ^S^	205	341 [M–H]^−^	1.86 ± 0.10	2.14 ± 0.12	26.18 ± 1.57
**2**	2.55	Glucose ^S^	205	179 [M–H]^−^	3.96 ± 0.22	6.29 ± 0.42	3.35 ± 0.17
**3**	2.78	Malic acid ^S^	205	133 [M–H]^−^	2.52 ± 0.15	4.27 ± 0.30	5.83 ± 0.36
**4**	3.04	Citric acid ^S^	205	191 [M–H]^−^	2.69 ± 0.18	4.83 ± 0.30	8.59 ± 0.50
**5**	3.66	2-Pyrone-4,6-dicarboxylic acid ^S^	314	183 [M–H]^−^; [183]→139 [(M–H)–CO_2_]^−^, 111 [(M–H)–COOH–CO]^−^	0.50 ± 0.03	1.29 ± 0.07	1.14 ± 0.06
**6**	3.72	Monogalloyl hexose ^L^	268	331 [M–H]^−^; [331]→169 [(M–H)–Hex]^−^	2.93 ± 0.16	1.54 ± 0.11	3.22 ± 0.19
**7**	4.93	Monogalloyl hexose ^L^	268	331 [M–H]^−^; [331]→169 [(M–H)–Hex]^−^	2.97 ± 0.18	2.83 ± 0.17	4.69 ± 0.28
**8**	5.42	Gallic acid ^S^	272	169 [M–H]^−^	0.29 ± 0.02	0.82 ± 0.06	0.80 ± 0.05
**9**	6.32	3,4-Dihydroxybenzoic acid 4-O-Glc ^S^	273	343 [M–H]^−^; [343]→181 [(M–H)–Glc]^−^	0.92 ± 0.06	1.63 ± 0.11	1.29 ± 0.08
**10**	6.64	*p*-Hydroxybenzoic acid *O*-hexoside isomer ^L^	274	299 [M–H]^−^; [299]→137 [(M–H)–Glc]^−^	0.83 ± 0.05	1.52 ± 0.09	1.16 ± 0.06
**11**	6.85	Digalloyl hexose ^L^	272	483 [M–H]^−^; [483]→331 [(M–H)–GalA]^−^	traces	traces	traces
**12**	7.04	Pedunculagin ^S^	270	783 [M–H]^−^; 391 [M–2H]^2−^	traces	traces	traces
**13**	8.14	1-*O*-*p*-Hydroxybenzoic acid *O*-Glc ^S^	274	299 [M–H]−; [299]→137 [(M–H)–Glc]−	1.16 ± 0.07	2.32 ± 0.15	2.09 ± 0.13
**14**	8.41	Digalloyl hexose ^L^	272	483 [M–H]−; [483]→331 [(M–H)–GalA]−	0.42 ± 0.02	0.29 ± 0.02	traces
**15**	8.59	Casuariin ^S^	270	783 [M–H]−; 391 [M–2H]2−	traces	traces	traces
**16**	9.34	Tellimagrandin I_1_^S^	272	785 [M–H]−; 392 [M–2H]2−; 1571 [2M–H]−	traces	0.53 ± 0.03	traces
**17**	9.77	Digalloyl hexose ^L^	272	483 [M–H]−; [483]→331 [(M–H)–GalA]−	0.51 ± 0.03	0.39 ± 0.03	0.10 ± 0.01
**18**	10.26	Trigalloyl hexose ^L^	274	635 [M–H]−; [635]→483 [(M–H)–GalA]−	0.59 ± 0.04	0.42 ± 0.02	0.24 ± 0.01
**19**	10.51	Trigalloyl hexose ^L^	274	635 [M–H]−; [635]→483 [(M–H)–GalA]−	0.78 ± 0.04	0.58 ± 0.04	0.43 ± 0.02
**20**	10.95	Trigalloyl hexose ^L^	274	635 [M–H]−; [635]→483 [(M–H)–GalA]−	2.58 ± 0.16	2.14 ± 0.14	2.03 ± 0.12
**21**	11.28	Tellimagrandin I_2_^S^	272	785 [M–H]−; 392 [M–2H]2−; 1571 [2M–H]−	0.52 ± 0.03	1.16 ± 0.07	1.10 ± 0.07
**22**	12.16	Trigalloyl hexose ^L^	274	635 [M–H]−; [635]→483 [(M–H)–Hex]−	3.56 ± 0.21	2.89 ± 0.20	2.72 ± 0.18
**23**	12.55	Trigalloyl-HHDP-Hex ^L^	273	951 [M–H]^−^; 475 [M–2H]^2−^	0.37 ± 0.02	0.26 ± 0.1	traces
**24**	13.38	Rugosin E_1_^S^	272	1721 [M–H]^−^; 860 [M–2H]^2−^	traces	0.50 ± 0.03	traces
**25**	13.47	Casuarinin ^S^	272	935 [M–H]^−^; 467 [M–2H]^2−^	traces	0.93 ± 0.06	traces
**26**	13.82	Rugosin E_2_^S^	272	1721 [M–H]^−^; 860 [M–2H]^2−^	traces	traces	traces
**27**	14.12	Potentillin ^S^	272	935 [M–H]^−^; 467 [M–2H]^2−^	traces	0.09 ± 0.01	traces
**28**	14.31	Casuarictin ^S^	276	937 [M–H]^−^; 468 [M–2H]^2−^	1.60 ± 0.09	5.28 ± 0.31	4.16 ± 0.28
**29**	14.66	Tetragalloyl hexose ^L^	271	787 [M–H]^−^; [787]→635 [(M–H)–GalA]^−^	3.14 ± 0.16	2.83 ± 0.15	0.83 ± 0.05
**30**	14.94	Agrimoniin ^S^	271	935 [M–H]^−^; 467 [M–2H]^2−^	traces	0.26 ± 0.02	traces
**31**	15.07	Tellimagrandin II_2_^S^	276	937 [M–H]^−^; 468 [M–2H]^2−^	3.62 ± 0.21	7.83 ± 0.51	5.16 ± 0.33
**32**	15.38	Kaempferol-3-*O*-Hex-Hex ^L^	265, 344	609 [M–H]^−^; [609]→447 [(M–H)–Glc]^−^, 285 [(M–H)–2×Glc]^−^	0.14 ± 0.01	0.52 ± 0.03	0.50 ± 0.03
**33**	15.49	Quercetin-3-*O*-GlcA ^S^	254, 268, 352	477 [M–H]−; [477]→301 [(M–H)–GlcA]−	7.88 ± 0.49	19.62 ± 1.20	12.84 ± 0.73
**34**	15.67	Pentagalloyl hexose ^L^	272	939 [M–H]−; [939]→787 [(M–H)–GalA]−	0.84 ± 0.05	0.52 ± 0.03	traces
**35**	15.88	Ellagic acid ^S^	255, 367	301 [M–H]−	traces	1.26 ± 0.08	3.18 ± 0.19
**36**	15.93	Digalloyl-bis-HHDP-Hex ^L^	271	1087 [M–H]−; 543 [M–2H]2−	0.17 ± 0.01	0.10 ± 0.01	traces
**37**	16.55	Kaempferol-3-*O*-GlcA ^S^	265, 344	461 [M–H]−; [461]→285 [(M–H)–GlcA]−	traces	3.22 ± 0.19	3.35 ± 0.20
**38**	16.76	Quercetin-7-*O*-Glc ^S^	254, 268, 364	463 [M–H]−; [463]→301 [(M–H)–Glc]−	0.26 ± 0.01	4.29 ± 0.26	4.35 ± 0.29
**39**	17.64	Quercetin-3-*O*-Ara ^S^	253, 268, 365	433 [M–H]−; [433]→301 [(M–H)–Ara]−	5.63 ± 0.33	21.59 ± 1.45	18.26 ± 1.06
**40**	18.59	Quercetin-3-*O*-(6″-*O*-Cin)-Glc ^S^	270, 285, 364	593 [M–H]−; [593]→463 [(M–H)–Cin]−, 301 [(M–H)–Glc]−	0.08 ± 0.01	1.53 ± 0.10	0.16 ± 0.01
**41**	25.42	Ellagic acid methyl ether ^L^	254, 362	315 [M–H]−; [315]→301 [(M–H)–CH2]−	0.18 ± 0.01	2.53 ± 0.15	3.19 ± 0.16

^a^ Compound identification was based on comparison of retention time and MS spectral data with reference standard (^S^) or interpretation of MS spectral data and comparison with literature data (^L^). Traces—<LOQ (limit of quantification). Abbreviation used: Glc—glucose; HHDP—hexahydroxyphenoyl; Hex—hexose; GlcA—glucuronic acid; GalA—gallic acid; Ara—arabinose; Cin—cinnamoyl.

## Data Availability

Data are contained within the article.

## Supplementary Materials

The following supporting information are available at: https://www.mdpi.com/article/10.3390/metabo13060689/s1, Table S1: Regression equations, correlation coefficients (r^2^), standard deviation (S_YX_), limits of detection (LOD), limits of quantification (LOQ), linear ranges, intra-day, inter-day precisions and recovery of spiked samples for 34 reference standards. Figure S1: Structures of compounds identified in *Geum aleppicum* and *Sibbaldianthe bifurca*.
