# Supplementary material for: Metabolites of Geum aleppicum and Sibbaldianthe bifurca: Diversity and α-Glucosidase Inhibitory Potential
Source: Metabolites. 2023 May 25;13(6):689. doi: 10.3390/metabo13060689 (PMC10304466; doi:10.3390/metabo13060689)
Supplement: Supplementary file 1 [file metabolites-13-00689-s001.zip › metabolites-2395829-supplementary.pdf]

Supplementary Material

Metabolites of *Geum aleppicum* and *Sibbaldianthe bifurca*: Diversity and α-Glucosidase Inhibitory Potential

Nina I. Kashchenko [1,*], Daniil N. Olennikov [1] and Nadezhda K. Chirikova [2]

[1] Laboratory of Medical and Biological Research, Institute of General and Experimental Biology, Siberian Division, Russian Academy of Science, 670047 Ulan-Ude, Russia
[2] Department of Biochemistry and Biotechnology, North-Eastern Federal University, 58 Belinsky Street, 677027 Yakutsk, Russia
* Correspondence: ninkk@mail.ru; Tel.: +8-983-421-73-40

Content:

Table S1. Regression equations, correlation coefficients (r^2), standard deviation (S_{YX}), limits of detection (LOD), limits of quantification (LOQ), linear ranges, intra-day, inter-day precisions and recovery of spiked samples for 34 reference standards.

Figure S1. Structures of compounds identified in *Geum aleppicum* and *Sibbaldianthe bifurca*.

Table S1. Regression equations, correlation coefficients (r^2), standard deviation (S_{YX}), limits of detection (LOD), limits of quantification (LOQ), linear ranges, intra-day, inter-day precisions and recovery of spiked samples for 34 reference standards.

Compound	CE [a] (eV)	Regression equation [b]		r^2	Syx	LOD/LOQ (μg/mL)	Linear Range (μg/mL)	RSD% (Intra-Day)	RSD% (Inter-Day)	Recovery of Spiked Sample REC%
		a	b·10 [6]							
Saccharose	-10	1.6278	-0.0428	0.9990	$7.11 \cdot 10^{-2}$	0.14/0.44	1.0–100.0	0.97	1.16	98.34
Glucose	-10	1.5632	-0.0376	0.9983	$5.14 \cdot 10^{-2}$	0.11/0.33	1.0–100.0	1.23	1.54	100.07
Malic acid	-10	0.9911	-0.0379	0.9988	$2.05 \cdot 10^{-2}$	0.07/0.21	1.0–100.0	1.09	1.36	99.12
Citric acid	-10	0.9518	-0.0267	0.9990	$1.03 \cdot 10^{-2}$	0.03/0.10	1.0–100.0	1.34	1.78	102.03
2-Pyrone-4,6-dicarboxylic acid	-10	0.9804	-0.0210	0.9970	$2.01 \cdot 10^{-2}$	0.06/0.21	1.0–100.0	1.47	1.64	100.78
Gallic acid	-20	2.6538	-0.1376	0.9990	$1.17 \cdot 10^{-2}$	0.01/0.04	1.0–100.0	1.22	1.43	101.22
3,4-Dihydroxybenzoic acid 4-*O*-Glc	-20	0.9214	-0.0373	0.9997	$2.10 \cdot 10^{-2}$	0.07/0.22	1.0–100.0	1.31	1.57	99.83
3,4,5-Trihydroxybenzaldehyde	-15	0.9320	-0.0523	0.9991	$4.14 \cdot 10^{-2}$	0.15/0.44	1.0–100.0	1.40	1.98	100.39
Pedunculagin	-35	0.6370	-0.4521	0.9872	$6.11 \cdot 10^{-2}$	0.32/0.96	1.0–100.0	0.99	1.60	102.55
1-*O*-*p*-Hydroxybenzoic acid *O*-Glc	-20	1.3586	-0.0663	0.9987	$9.69 \cdot 10^{-2}$	0.24/0.71	1.0–100.0	1.38	1.88	101.14
Casuariin	-20	1.0634	-0.0933	0.9902	$10.01 \cdot 10^{-2}$	0.31/0.94	1.0–100.0	0.89	1.29	100.64
Tellimagrandin I_1	-35	0.9634	-0.3518	0.9804	$7.34 \cdot 10^{-2}$	0.25/0.76	1.0–100.0	1.12	1.42	99.23
Tellimagrandin I_2	-35	1.4267	-0.5637	0.9907	$12.72 \cdot 10^{-2}$	0.29/0.89	1.0–100.0	1.33	1.61	100.11
Rugosin E_1	-20	1.6341	-0.4283	0.9900	$15.02 \cdot 10^{-2}$	0.30/0.92	1.0–100.0	1.45	1.92	101.27
Casuarinin	-20	0.9634	-0.8634	0.9832	$10.37 \cdot 10^{-2}$	0.36/1.07	1.0–100.0	1.29	1.43	98.26
Rugosin E_2	-20	1.4238	-0.0891	0.9901	$7.33 \cdot 10^{-2}$	0.17/0.52	1.0–100.0	1.15	1.39	99.30
Potentillin	-20	1.5152	-0.0523	0.9979	$12.67 \cdot 10^{-2}$	0.28/0.84	1.0–100.0	1.28	1.66	100.78
Casuarictin	-20	0.8237	-0.7310	0.9801	$14.73 \cdot 10^{-2}$	0.59/1.79	1.0–100.0	1.36	1.75	101.33
Agrimoniin	-35	0.8214	-0.2716	0.9893	$5.37 \cdot 10^{-2}$	0.22/0.65	1.0–100.0	1.44	1.84	102.00

Gemin A	-35	1.4412	-0.6211	0.9930	$11.25 \cdot 10^{-2}$	0.26/0.78	1.0–100.0	1.39	1.90	100.36
Tellimagrandin II_2	-35	0.9361	-0.4518	0.9870	$9.35 \cdot 10^{-2}$	0.32/1.00	1.0–100.0	1.42	1.73	101.17
Quercetin-3-*O*-GlcA	-20	1.6705	-0.4374	0.9988	$12.79 \cdot 10^{-2}$	0.25/0.77	1.0–100.0	1.21	1.48	102.08
Quercetin-3-*O*-Glc	-20	1.1103	-0.9217	0.9901	$14.33 \cdot 10^{-2}$	0.42/1.29	1.0–100.0	1.08	1.55	99.39
Quercetin-7-*O*-Glc	-20	1.2703	-0.7911	0.9814	$15.26 \cdot 10^{-2}$	0.40/1.20	1.0–100.0	1.14	1.69	101.21
Quercetin-3-*O*-Ara	-20	1.4412	-0.6211	0.9930	$11.25 \cdot 10^{-2}$	0.26/0.78	1.0–100.0	1.25	1.58	102.05
Quercetin-3-*O*-(6′′-*O*-Cin)-Glc	-20	1.2703	-0.7911	0.9814	$15.26 \cdot 10^{-2}$	0.40/1.20	1.0–100.0	1.30	2.01	99.32
Ellagic acid	-30	0.9114	-0.6312	0.9887	$6.37 \cdot 10^{-2}$	0.23/0.70	1.0–100.0	1.16	1.97	100.84
Kaempferol-3-*O*-Glc	-20	2.0859	-0.9171	0.9980	$6.18 \cdot 10^{-2}$	0.03/0.09	1.0–100.0	1.23	1.83	101.23
Kaempferol-3-*O*-GlcA	-30	2.2126	-0.5160	0.9987	$8.11 \cdot 10^{-2}$	0.12/0.37	1.0–100.0	1.08	1.60	98.33
Niga-ichigoside F1	-30	2.0319	-0.3615	0.9811	$10.09 \cdot 10^{-2}$	0.17/0.52	1.0–100.0	1.05	1.36	100.50
Rosamultin (tormentic acid *O*-Glc)	-30	1.5364	-0.3614	0.9927	$10.07 \cdot 10^{-2}$	0.22/0.66	1.0–100.0	1.12	1.44	102.13
Tormentic acid	-30	1.5330	-0.0863	0.9985	$4.15 \cdot 10^{-2}$	0.09/0.27	1.0–100.0	1.39	1.78	100.09
Corosolic acid	-30	2.3312	-0.4563	0.9803	$14.92 \cdot 10^{-2}$	0.21/0.64	1.0–100.0	1.24	1.85	101.40
Ursolic acid	-30	1.2820	-0.9634	0.9697	$11.64 \cdot 10^{-2}$	0.30/0.91	1.0–100.0	1.41	1.91	99.54

[a] CE – collision energy. [b] Regression equation: $y = a \cdot x + b$.

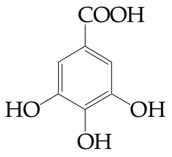

Gallic acid

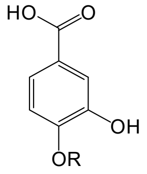

3,4-Dihydroxybenzoic acid 4-*O*-Glc: R = Glc

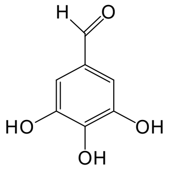

3,4,5-Trihydroxybenzaldehyde

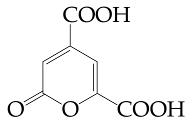

2-Pyrone-4,6-dicarboxylic acid

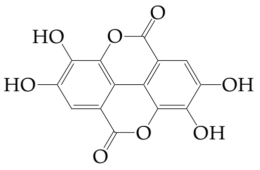

Ellagic acid

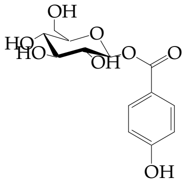

1-*O*-*p*-Hydroxybenzoyl glucose

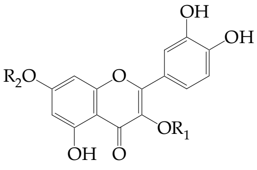

Quercetin-3-*O*-GlcA: R_1 = GlcA; R_2 = H

Quercetin-3-*O*-Glc: R_1 = Glc; R_2 = H

Quercetin-3-*O*-Ara: R_1 = Ara; R_2 = H

Quercetin-3-*O*-(6′′-*O*-Cin)-Glc: R_1 = (6′′-*O*-Cin)-Glc; R_2 = H

Quercetin-7-*O*-Glc: R_1 = H; R_2 = Glc

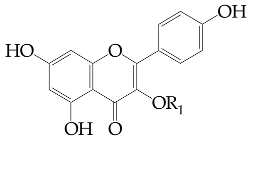

Kaempferol-3-*O*-GlcA: R_1 = GlcA

Kaempferol-3-*O*-Glc: R_1 = Glc

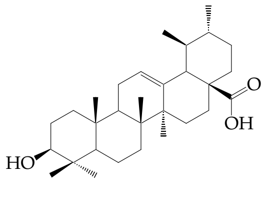

Ursolic acid

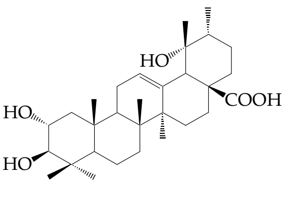

Tormentic acid

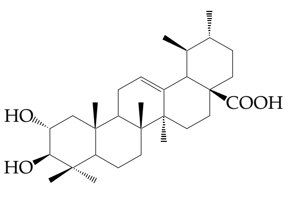

Corosolic acid

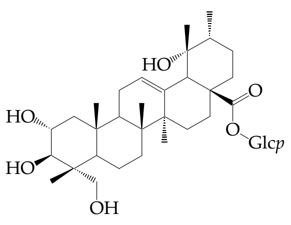

Niga-ichigoside F_1

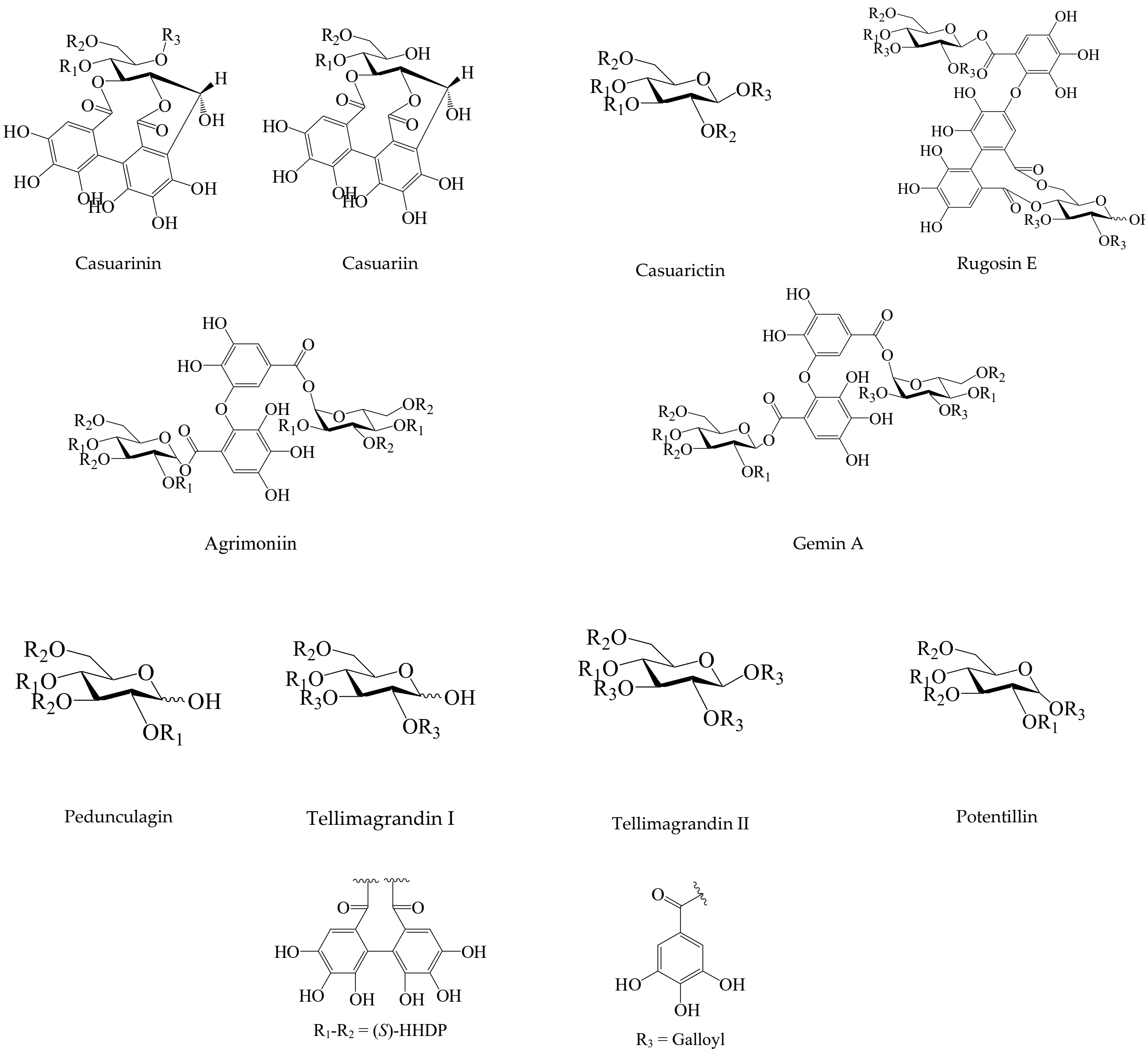

Figure S1. Structures of compounds identified in *Geum aleppicum* and *Sibbaldianthe bifurca*. Abbreviation used: Glc – glucose; GlcA – glucuronic acid; Ara – arabinose; Cin – cinnamoyl; HHDP – hexahydroxydiphenoyl.
